# 3-(4-Hexyl­oxyphen­yl)-1,2,4-triazolo[3,4-*b*]benzo­thia­zole

**DOI:** 10.1107/S1600536814002153

**Published:** 2014-02-05

**Authors:** Dieter Schollmeyer, Heiner Detert

**Affiliations:** a University Mainz, Duesbergweg 10-14, 55099 Mainz, Germany

## Abstract

The title compound, C_20_H_21_N_3_OS, was prepared by Huisgen reaction of 5-(4-hexyl­oxyphen­yl)tetra­zole and chloro­benzo­thia­zole. The essentially planar benzo­thia­zolotriazole framework [maximum deviation from the mean plane of 0.077 (1) Å for the bridgehead N atom] and the phenyl ring form a dihedral angle of 53.34 (5)°. The hex­yloxy chain adopts a *gauche*–*all*-anti conformation. The intra­centroid separation of 3.7258 (8) Å between the triazole and benzene rings is the closest contact between individual mol­ecules in the crystal.

## Related literature   

For related benzo­thia­zolotriazoles, see: Butler *et al.* (1972 ▶); Reynolds & van Allan (1959 ▶). For triazolo-annulation *via* tetra­zoles, see: Christiano *et al.* (2008 ▶). For the Huisgen reaction, see: Huisgen *et al.* (1960 ▶,1961 ▶). For the structures of related triazolo-annulated heterocycles, see: Preis *et al.* (2011*a* ▶,*b* ▶); Herget *et al.* (2013 ▶); Puviarasnan *et al.* (1999 ▶).
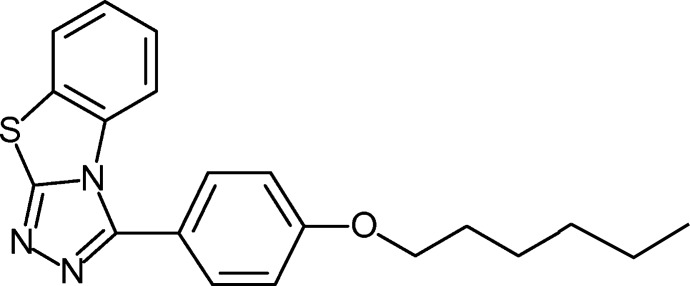


## Experimental   

### 

#### Crystal data  

C_20_H_21_N_3_OS
*M* *_r_* = 351.46
Orthorhombic, 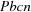

*a* = 10.7369 (4) Å
*b* = 9.1770 (3) Å
*c* = 35.6567 (11) Å
*V* = 3513.3 (2) Å^3^
*Z* = 8
Mo *K*α radiation
μ = 0.20 mm^−1^
*T* = 193 K
0.50 × 0.20 × 0.20 mm

#### Data collection  

Stoe IPDS 2T diffractometer
26869 measured reflections
4225 independent reflections
3441 reflections with *I* > 2σ(*I*)
*R*_int_ = 0.074

#### Refinement  

*R*[*F* ^2^ > 2σ(*F* ^2^)] = 0.047
*wR*(*F* ^2^) = 0.119
*S* = 1.04
4225 reflections
227 parameters
H-atom parameters constrained
Δρ_max_ = 0.30 e Å^−3^
Δρ_min_ = −0.23 e Å^−3^

### 

Data collection: *X-AREA* (Stoe & Cie, 2011 ▶); cell refinement: *X-AREA*; data reduction: *X-RED* (Stoe & Cie, 2011 ▶); program(s) used to solve structure: *SIR97* (Altomare *et al.*, 1999 ▶); program(s) used to refine structure: *SHELXL97* (Sheldrick, 2008 ▶); molecular graphics: *PLATON* (Spek, 2009 ▶); software used to prepare material for publication: *PLATON*.

## Supplementary Material

Crystal structure: contains datablock(s) I, New_Global_Publ_Block. DOI: 10.1107/S1600536814002153/nc2323sup1.cif

Structure factors: contains datablock(s) I. DOI: 10.1107/S1600536814002153/nc2323Isup2.hkl

Supporting information file. DOI: 10.1107/S1600536814002153/nc2323Isup3.cml

CCDC reference:

Additional supporting information:  crystallographic information; 3D view; checkCIF report

## Figures and Tables

**Fig. 1.**
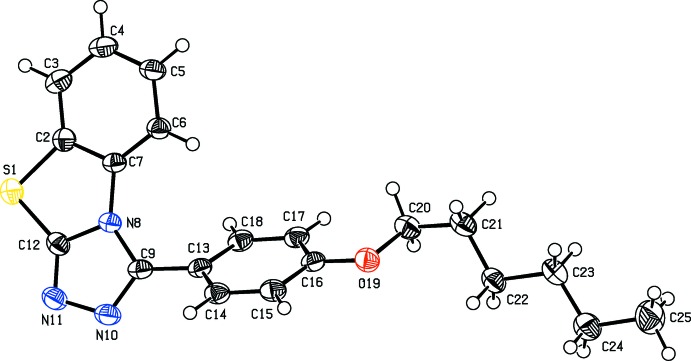
Crystal structure of the title compound with labeling and displacement ellipsoids drawn at the 50% probability level.

## supplementary crystallographic information

### 1. Comment

The Huisgen reaction of tetrazoles and 2-chloroazines is a versatile method for the synthesis of 1,2,4-triazolo-annulated azines see: Preis *et al.* (2011*a*, 2011*b*), Herget *et al.* (2013), and Christiano *et al.* (2008). This method is also applicable to azoles with active chlorine and this structure confirms the corresponding [3,4-*b*]- annulation. The essentially planar benzothiazolotriazole framework of title compound C_20_H_21_N_3_OS (max. deviation 0.077 Å from mean plane at N(8) and the planar phenyl ring open a dihedral angle of 53.34 (5)°. The structural features of the π-system are very similar to a derivative lacking the hexyloxy group, see Puviarasnan *et al.* (1999). The O19—C20 bond is nearly coplanar with the mean plane of the phenyl ring (torsion angle: 6.6°) and the hexyl chain shows a *gauche*-*all*-anti konformation. A minimal distance of 3.73 Å between neighbouring molecules was found for the centroids of the triazole and the benzo-ring.

### 2. Experimental

The title compound was prepared by adding chlorobenzothiazole (0.49 g, 2.75 mmol) to a stirred solution of 5-(4-hexyloxyphenyl)tetrazole (0.67 g, 2.75 mmol) and collidine (0.5 ml) in xylenes (12 ml). The mixture was stirred for 15 h at ambient temperature and heated to 392 K for 24 h. The cooled solution was filtered, washed with water (30 ml), dried (CaCl_2_) and concentrated. Chromatography on silica gel using toluene / ethyl acetate 3 / 7 (*R*_f_ = 0.15) as an eluent yielded 0.66 g of the pure title compound (68%). Recrystallization from methanol / dichloromethane gave off-white crystals with m.p. = 400 K

### 3. Refinement

Hydrogen atoms attached to carbons were placed at calculated positions with C—H = 0.95 Å (aromatic) or 0.98–0.99 Å (*sp*^3^ C-atom). All H atoms were refined in the riding-model approximation with isotropic displacement parameters (set at 1.2–1.5 times of the *U*_eq_ of the parent atom).

### Crystal data

**Table:**

C_20_H_21_N_3_OS	*D*_x_ = 1.329 Mg m^−^^3^
*M**_r_* = 351.46	Melting point: 400 K
Orthorhombic, *P**b**c**n*	Mo *K*α radiation, λ = 0.71073 Å
Hall symbol: -P 2n 2ab	Cell parameters from 32877 reflections
*a* = 10.7369 (4) Å	θ = 2.2–33.7°
*b* = 9.1770 (3) Å	µ = 0.20 mm^−^^1^
*c* = 35.6567 (11) Å	*T* = 193 K
*V* = 3513.3 (2) Å^3^	Needle, colourless
*Z* = 8	0.50 × 0.20 × 0.20 mm
*F*(000) = 1488	

### Data collection

**Table:**

Stoe IPDS 2T diffractometer	3441 reflections with *I* > 2σ(*I*)
Radiation source: sealed X-ray tube, 12 x 0.4 mm long-fine focus	*R*_int_ = 0.074
Graphite monochromator	θ_max_ = 28.0°, θ_min_ = 2.9°
Detector resolution: 6.67 pixels mm^-1^	*h* = −14→14
rotation method scans	*k* = −8→12
26869 measured reflections	*l* = −47→38
4225 independent reflections	

### Refinement

**Table:**

Refinement on *F*^2^	Primary atom site location: structure-invariant direct methods
Least-squares matrix: full	Secondary atom site location: difference Fourier map
*R*[*F*^2^ > 2σ(*F*^2^)] = 0.047	Hydrogen site location: inferred from neighbouring sites
*wR*(*F*^2^) = 0.119	H-atom parameters constrained
*S* = 1.04	*w* = 1/[σ^2^(*F*_o_^2^) + (0.0653*P*)^2^ + 0.9425*P*] where *P* = (*F*_o_^2^ + 2*F*_c_^2^)/3
4225 reflections	(Δ/σ)_max_ = 0.001
227 parameters	Δρ_max_ = 0.30 e Å^−^^3^
0 restraints	Δρ_min_ = −0.23 e Å^−^^3^

### Special details

**Table:**

Experimental. ^1^H-NMR (CDCl_3_): δ = 7.66 (m, 3 H); 7.51 (d, 1 H, J = 7.5 Hz), 7.35 ("t", 1 H), 7.29 ("t", 1H), 7.06 (d, 2 H, J = 8 Hz), 4.05, t, 2 H), 1.83 (qui, 2 H), 1.48 (qui, 2 H), 1.33 (m, 4 H), 0.90 ("t", 3H). ^13^C-NMR(CDCl_3_): δ = 156.0, 150.7, 154.3, 127.5, 125.5 (2c), 125.0, 121.2, 121.1, 119.7, 113.5, 109.9, 109.2, 63.2, 26.5, 24.1, 20.6, 17.5, 8.9. FD—MS:351.4 (*M*+H^+^). UV-Vis: dichloromethane: λmax = 260 nm (log ε = 4.11), λmax = 298 nm (log ε = 3.85); cyclohexane: λmax = 261 nm, λmax = 298 nm; λmax = 298 nm; ethanol: λmax = 258 nm, λmax = 295 nm; fluorescence: dichloromethane: λmax = 356 nm; cyclohexane: λmax = 333 nm; ethanol: λmax = 358 nm.
Geometry. All e.s.d.'s (except the e.s.d. in the dihedral angle between two l.s. planes) are estimated using the full covariance matrix. The cell e.s.d.'s are taken into account individually in the estimation of e.s.d.'s in distances, angles and torsion angles; correlations between e.s.d.'s in cell parameters are only used when they are defined by crystal symmetry. An approximate (isotropic) treatment of cell e.s.d.'s is used for estimating e.s.d.'s involving l.s. planes.
Refinement. Refinement of *F*^2^ against ALL reflections. The weighted *R*-factor *wR* and goodness of fit *S* are based on *F*^2^, conventional *R*-factors *R* are based on *F*, with *F* set to zero for negative *F*^2^. The threshold expression of *F*^2^ > σ(*F*^2^) is used only for calculating *R*-factors(gt) *etc*. and is not relevant to the choice of reflections for refinement. *R*-factors based on *F*^2^ are statistically about twice as large as those based on *F*, and *R*- factors based on ALL data will be even larger.

### Fractional atomic coordinates and isotropic or equivalent isotropic displacement parameters (Å^2^)

**Table:**

	*x*	*y*	*z*	*U*_iso_*/*U*_eq_	
S1	0.41618 (4)	0.26300 (4)	0.696695 (10)	0.03581 (13)	
C2	0.40821 (12)	0.43744 (15)	0.67607 (4)	0.0304 (3)	
C3	0.41068 (14)	0.57003 (18)	0.69481 (4)	0.0364 (3)	
H3	0.4124	0.5737	0.7214	0.044*	
C4	0.41053 (13)	0.69677 (17)	0.67376 (5)	0.0374 (3)	
H4	0.4115	0.7884	0.6861	0.045*	
C5	0.40897 (13)	0.69200 (16)	0.63482 (5)	0.0340 (3)	
H5	0.4109	0.7804	0.6210	0.041*	
C6	0.40465 (12)	0.55988 (15)	0.61587 (4)	0.0299 (3)	
H6	0.4030	0.5564	0.5892	0.036*	
C7	0.40282 (11)	0.43353 (14)	0.63696 (4)	0.0260 (3)	
N8	0.40149 (10)	0.28738 (12)	0.62443 (3)	0.0263 (2)	
C9	0.40200 (12)	0.20746 (15)	0.59169 (4)	0.0280 (3)	
N10	0.41399 (11)	0.06852 (13)	0.60013 (4)	0.0348 (3)	
N11	0.42111 (12)	0.05377 (13)	0.63912 (4)	0.0361 (3)	
C12	0.41293 (12)	0.18574 (15)	0.65223 (4)	0.0304 (3)	
C13	0.38612 (12)	0.26957 (15)	0.55412 (4)	0.0280 (3)	
C14	0.46740 (13)	0.23437 (15)	0.52481 (4)	0.0311 (3)	
H14	0.5331	0.1670	0.5291	0.037*	
C15	0.45328 (13)	0.29627 (16)	0.48985 (4)	0.0323 (3)	
H15	0.5089	0.2710	0.4702	0.039*	
C16	0.35734 (12)	0.39615 (15)	0.48326 (4)	0.0289 (3)	
C17	0.27419 (12)	0.42904 (15)	0.51209 (4)	0.0307 (3)	
H17	0.2073	0.4947	0.5077	0.037*	
C18	0.28918 (12)	0.36596 (15)	0.54696 (4)	0.0304 (3)	
H18	0.2321	0.3889	0.5664	0.036*	
O19	0.35160 (9)	0.45394 (11)	0.44817 (3)	0.0345 (2)	
C20	0.25760 (14)	0.56334 (16)	0.44224 (4)	0.0354 (3)	
H20A	0.2658	0.6407	0.4614	0.043*	
H20B	0.1739	0.5191	0.4448	0.043*	
C21	0.27167 (14)	0.62833 (16)	0.40378 (4)	0.0367 (3)	
H21A	0.3565	0.6698	0.4016	0.044*	
H21B	0.2118	0.7098	0.4013	0.044*	
C22	0.25134 (13)	0.52382 (17)	0.37123 (4)	0.0349 (3)	
H22A	0.3231	0.4564	0.3695	0.042*	
H22B	0.1757	0.4650	0.3760	0.042*	
C23	0.23673 (14)	0.60407 (17)	0.33430 (4)	0.0368 (3)	
H23A	0.1652	0.6716	0.3364	0.044*	
H23B	0.3123	0.6635	0.3299	0.044*	
C24	0.21655 (17)	0.50588 (19)	0.30070 (5)	0.0451 (4)	
H24A	0.2927	0.4472	0.2965	0.054*	
H24B	0.1475	0.4377	0.3063	0.054*	
C25	0.1863 (2)	0.5888 (2)	0.26520 (5)	0.0612 (5)	
H25A	0.1085	0.6430	0.2687	0.092*	
H25B	0.1768	0.5200	0.2444	0.092*	
H25C	0.2540	0.6570	0.2596	0.092*	

### Atomic displacement parameters (Å^2^)

**Table:**

	*U*^11^	*U*^22^	*U*^33^	*U*^12^	*U*^13^	*U*^23^
S1	0.0409 (2)	0.0353 (2)	0.0313 (2)	0.00007 (15)	−0.00034 (14)	0.00440 (14)
C2	0.0281 (6)	0.0311 (7)	0.0319 (7)	0.0002 (5)	−0.0004 (5)	−0.0004 (6)
C3	0.0350 (7)	0.0393 (8)	0.0350 (8)	−0.0002 (6)	−0.0015 (6)	−0.0103 (6)
C4	0.0349 (7)	0.0303 (7)	0.0471 (9)	0.0004 (6)	−0.0036 (6)	−0.0129 (7)
C5	0.0333 (7)	0.0239 (7)	0.0449 (8)	−0.0005 (5)	−0.0033 (6)	−0.0039 (6)
C6	0.0291 (6)	0.0258 (7)	0.0348 (7)	−0.0019 (5)	−0.0016 (5)	−0.0013 (6)
C7	0.0228 (6)	0.0231 (6)	0.0322 (7)	−0.0009 (5)	0.0001 (5)	−0.0043 (5)
N8	0.0271 (5)	0.0215 (5)	0.0302 (6)	−0.0011 (4)	0.0003 (4)	−0.0009 (4)
C9	0.0250 (6)	0.0237 (6)	0.0353 (7)	−0.0015 (5)	−0.0006 (5)	−0.0060 (5)
N10	0.0345 (6)	0.0243 (6)	0.0455 (7)	−0.0005 (5)	−0.0050 (5)	−0.0035 (5)
N11	0.0385 (6)	0.0255 (6)	0.0443 (7)	−0.0007 (5)	−0.0040 (5)	0.0030 (5)
C12	0.0283 (6)	0.0267 (7)	0.0360 (7)	−0.0010 (5)	−0.0005 (5)	0.0041 (6)
C13	0.0275 (6)	0.0249 (6)	0.0315 (7)	−0.0025 (5)	−0.0028 (5)	−0.0063 (5)
C14	0.0270 (6)	0.0281 (7)	0.0382 (7)	0.0043 (5)	−0.0008 (6)	−0.0067 (6)
C15	0.0304 (7)	0.0306 (7)	0.0358 (7)	0.0030 (5)	0.0038 (6)	−0.0060 (6)
C16	0.0286 (6)	0.0257 (6)	0.0324 (7)	−0.0016 (5)	−0.0025 (5)	−0.0061 (5)
C17	0.0267 (6)	0.0292 (7)	0.0361 (7)	0.0037 (5)	−0.0033 (5)	−0.0085 (6)
C18	0.0264 (6)	0.0314 (7)	0.0334 (7)	0.0008 (5)	0.0008 (5)	−0.0077 (6)
O19	0.0360 (5)	0.0331 (5)	0.0343 (5)	0.0066 (4)	0.0009 (4)	−0.0016 (4)
C20	0.0336 (7)	0.0292 (7)	0.0435 (8)	0.0034 (5)	0.0009 (6)	0.0006 (6)
C21	0.0346 (7)	0.0274 (7)	0.0482 (8)	−0.0013 (6)	0.0007 (6)	0.0057 (6)
C22	0.0319 (7)	0.0290 (7)	0.0437 (8)	−0.0024 (5)	−0.0040 (6)	0.0081 (6)
C23	0.0369 (7)	0.0303 (7)	0.0431 (8)	0.0024 (6)	0.0039 (6)	0.0081 (6)
C24	0.0515 (9)	0.0386 (9)	0.0452 (9)	−0.0031 (7)	0.0014 (7)	0.0053 (7)
C25	0.0812 (14)	0.0626 (12)	0.0398 (9)	0.0116 (11)	0.0092 (9)	0.0047 (9)

### Geometric parameters (Å, º)

**Table:**

S1—C12	1.7370 (15)	C16—O19	1.3605 (17)
S1—C2	1.7638 (15)	C16—C17	1.395 (2)
C2—C3	1.388 (2)	C17—C18	1.381 (2)
C2—C7	1.396 (2)	C17—H17	0.9500
C3—C4	1.384 (2)	C18—H18	0.9500
C3—H3	0.9500	O19—C20	1.4392 (17)
C4—C5	1.389 (2)	C20—C21	1.503 (2)
C4—H4	0.9500	C20—H20A	0.9900
C5—C6	1.389 (2)	C20—H20B	0.9900
C5—H5	0.9500	C21—C22	1.521 (2)
C6—C7	1.382 (2)	C21—H21A	0.9900
C6—H6	0.9500	C21—H21B	0.9900
C7—N8	1.4137 (17)	C22—C23	1.517 (2)
N8—C12	1.3667 (18)	C22—H22A	0.9900
N8—C9	1.3787 (18)	C22—H22B	0.9900
C9—N10	1.3164 (18)	C23—C24	1.515 (2)
C9—C13	1.466 (2)	C23—H23A	0.9900
N10—N11	1.399 (2)	C23—H23B	0.9900
N11—C12	1.3012 (19)	C24—C25	1.512 (2)
C13—C18	1.3897 (19)	C24—H24A	0.9900
C13—C14	1.399 (2)	C24—H24B	0.9900
C14—C15	1.378 (2)	C25—H25A	0.9800
C14—H14	0.9500	C25—H25B	0.9800
C15—C16	1.399 (2)	C25—H25C	0.9800
C15—H15	0.9500		
			
C12—S1—C2	89.37 (7)	C18—C17—H17	120.1
C3—C2—C7	120.26 (13)	C16—C17—H17	120.1
C3—C2—S1	126.43 (12)	C17—C18—C13	121.31 (13)
C7—C2—S1	113.28 (11)	C17—C18—H18	119.3
C4—C3—C2	118.38 (14)	C13—C18—H18	119.3
C4—C3—H3	120.8	C16—O19—C20	116.03 (11)
C2—C3—H3	120.8	O19—C20—C21	109.90 (12)
C3—C4—C5	121.03 (14)	O19—C20—H20A	109.7
C3—C4—H4	119.5	C21—C20—H20A	109.7
C5—C4—H4	119.5	O19—C20—H20B	109.7
C6—C5—C4	120.94 (14)	C21—C20—H20B	109.7
C6—C5—H5	119.5	H20A—C20—H20B	108.2
C4—C5—H5	119.5	C20—C21—C22	115.56 (12)
C7—C6—C5	117.91 (14)	C20—C21—H21A	108.4
C7—C6—H6	121.0	C22—C21—H21A	108.4
C5—C6—H6	121.0	C20—C21—H21B	108.4
C6—C7—C2	121.42 (13)	C22—C21—H21B	108.4
C6—C7—N8	128.61 (13)	H21A—C21—H21B	107.5
C2—C7—N8	109.90 (12)	C23—C22—C21	111.77 (13)
C12—N8—C9	104.52 (12)	C23—C22—H22A	109.3
C12—N8—C7	114.67 (12)	C21—C22—H22A	109.3
C9—N8—C7	140.56 (12)	C23—C22—H22B	109.3
N10—C9—N8	108.80 (13)	C21—C22—H22B	109.3
N10—C9—C13	126.64 (13)	H22A—C22—H22B	107.9
N8—C9—C13	124.51 (12)	C24—C23—C22	114.37 (13)
C9—N10—N11	109.03 (12)	C24—C23—H23A	108.7
C12—N11—N10	105.28 (12)	C22—C23—H23A	108.7
N11—C12—N8	112.37 (13)	C24—C23—H23B	108.7
N11—C12—S1	134.96 (12)	C22—C23—H23B	108.7
N8—C12—S1	112.65 (10)	H23A—C23—H23B	107.6
C18—C13—C14	118.47 (13)	C25—C24—C23	113.19 (15)
C18—C13—C9	120.17 (12)	C25—C24—H24A	108.9
C14—C13—C9	121.35 (12)	C23—C24—H24A	108.9
C15—C14—C13	120.78 (13)	C25—C24—H24B	108.9
C15—C14—H14	119.6	C23—C24—H24B	108.9
C13—C14—H14	119.6	H24A—C24—H24B	107.8
C14—C15—C16	120.21 (13)	C24—C25—H25A	109.5
C14—C15—H15	119.9	C24—C25—H25B	109.5
C16—C15—H15	119.9	H25A—C25—H25B	109.5
O19—C16—C17	124.37 (12)	C24—C25—H25C	109.5
O19—C16—C15	116.32 (12)	H25A—C25—H25C	109.5
C17—C16—C15	119.31 (13)	H25B—C25—H25C	109.5
C18—C17—C16	119.89 (12)		
			
C12—S1—C2—C3	−177.71 (13)	C7—N8—C12—N11	−174.91 (11)
C12—S1—C2—C7	0.27 (10)	C9—N8—C12—S1	179.33 (9)
C7—C2—C3—C4	−1.4 (2)	C7—N8—C12—S1	3.92 (14)
S1—C2—C3—C4	176.40 (11)	C2—S1—C12—N11	176.13 (15)
C2—C3—C4—C5	−0.5 (2)	C2—S1—C12—N8	−2.34 (10)
C3—C4—C5—C6	1.5 (2)	N10—C9—C13—C18	128.50 (15)
C4—C5—C6—C7	−0.4 (2)	N8—C9—C13—C18	−48.64 (19)
C5—C6—C7—C2	−1.54 (19)	N10—C9—C13—C14	−51.7 (2)
C5—C6—C7—N8	−178.36 (12)	N8—C9—C13—C14	131.13 (14)
C3—C2—C7—C6	2.5 (2)	C18—C13—C14—C15	1.4 (2)
S1—C2—C7—C6	−175.59 (10)	C9—C13—C14—C15	−178.40 (13)
C3—C2—C7—N8	179.88 (12)	C13—C14—C15—C16	0.3 (2)
S1—C2—C7—N8	1.76 (13)	C14—C15—C16—O19	178.83 (13)
C6—C7—N8—C12	173.48 (13)	C14—C15—C16—C17	−1.9 (2)
C2—C7—N8—C12	−3.63 (15)	O19—C16—C17—C18	−179.06 (12)
C6—C7—N8—C9	0.5 (2)	C15—C16—C17—C18	1.7 (2)
C2—C7—N8—C9	−176.62 (14)	C16—C17—C18—C13	0.0 (2)
C12—N8—C9—N10	−0.47 (14)	C14—C13—C18—C17	−1.6 (2)
C7—N8—C9—N10	172.96 (14)	C9—C13—C18—C17	178.22 (12)
C12—N8—C9—C13	177.11 (12)	C17—C16—O19—C20	4.47 (19)
C7—N8—C9—C13	−9.5 (2)	C15—C16—O19—C20	−176.25 (12)
N8—C9—N10—N11	0.29 (15)	C16—O19—C20—C21	173.79 (12)
C13—C9—N10—N11	−177.22 (12)	O19—C20—C21—C22	63.84 (16)
C9—N10—N11—C12	0.02 (15)	C20—C21—C22—C23	166.39 (12)
N10—N11—C12—N8	−0.33 (15)	C21—C22—C23—C24	179.82 (13)
N10—N11—C12—S1	−178.80 (12)	C22—C23—C24—C25	172.79 (15)
C9—N8—C12—N11	0.50 (15)		
